# Male Sex and the Risk of Childhood Cancer: The Mediating Effect of Birth Defects

**DOI:** 10.1093/jncics/pkaa052

**Published:** 2020-06-11

**Authors:** Erin L Marcotte, Jeremy M Schraw, Tania A Desrosiers, Wendy N Nembhard, Peter H Langlois, Mark A Canfield, Robert E Meyer, Sharon E Plon, Philip J Lupo

**Affiliations:** p1 Department of Pediatrics, University of Minnesota, Minneapolis, MN, USA; p2 Masonic Cancer Center, University of Minnesota, Minneapolis, MN, USA; p3 Department of Medicine, Baylor College of Medicine, Houston, TX, USA; p4 Department of Epidemiology, University of North Carolina, Chapel Hill, NC, USA; p5 Department of Epidemiology, University of Arkansas for Medical Sciences, and Arkansas Children’s Research Institute, Little Rock, AR, USA; p6 Texas Department, of State Health Services, Austin, TX, USA; p7 Department of Maternal and Child Health, University of North Carolina, Chapel Hill, NC, USA; p8 Department of Pediatrics, Baylor College of Medicine, Houston, TX, USA; p9 Texas Children’s Cancer Center, Texas Children’s Hospital, Houston, TX, USA

## Abstract

**Background:**

There is a persistent, unexplained disparity in sex ratio among childhood cancer cases, whereby males are more likely to develop most cancers. This male predominance is also seen for most birth defects, which are strongly associated with risk of childhood cancer. We conducted mediation analysis to estimate whether the increased risk of cancer among males is partially explained by birth defect status.

**Methods:**

We used a population-based birth cohort with linked data from birth certificates, birth defects registries, and cancer registries from Arkansas, Michigan, North Carolina, and Texas. We conducted counterfactual mediation analysis to estimate the natural direct and indirect effects of sex on cancer risk, modeling birth defect status as mediator. State; birth year; plurality; and maternal race and ethnicity, age, and education were considered confounders. We conducted separate analyses limited to cancers diagnosed younger than 1 year of age.

**Results:**

Our dataset included 10 181 074 children: 15 110 diagnosed with cancer, 539 567 diagnosed with birth defects, and 2124 co-occurring cases. Birth defect status mediated 38% of the association between sex and cancer overall. The proportion mediated varied by cancer type, including acute myeloid leukemia (93%), neuroblastoma (35%), and non-Hodgkin lymphoma (6%). Among children younger than 1 year of age at cancer diagnosis, the proportion mediated was substantially higher (82%).

**Conclusions:**

Our results suggest that birth defects mediate a statistically significant proportion of the relationship between sex and childhood cancer. The proportion mediated varied by cancer type and diagnosis age. These findings improve our understanding of the causal pathway underlying male sex as a risk factor for childhood cancer.

It is well established that males have a higher risk of childhood cancer compared with females (1). This male excess in incidence is observed for most major tumor types and the overall male-to-female incidence rate ratio for all childhood cancers is 1.19 (95% confidence interval [CI] = 1.18 to 1.20) (1), with a range for specific cancers from 1.13 (astrocytoma and neuroblastoma) to 4.62 (Burkitt lymphoma). The tumors with a female preponderance are nephroblastoma (Wilms tumor), extracranial and extragonadal germ cell tumors, and thyroid carcinoma. The causal pathways underlying the association between male sex and childhood cancer are unknown.

Birth defects have emerged as another important risk factor for childhood cancer (2–8). Increased risk for childhood cancers has been observed for children with chromosomal anomalies and monogenic syndromes and those with nonsyndromic birth defects. This association is consistent across nearly every major tumor type, which was confirmed in a recent analysis that used the dataset described herein (2).

These associations are notable, as there is evidence that males are also more likely to be born with a birth defect. A report from the National Birth Defects Prevention Study described a male-to-female sex ratio of 1.18 (95% CI = 1.13 to 1.24) among 25 952 clinically reviewed infants with a documented birth defect (9). Anomalies with a high male preponderance included craniosynostosis (sex ratio = 2.05, 95% CI = 1.81 to 2.32), left ventricular outflow tract defects (sex ratio = 2.20, 95% CI = 1.94 to 2.49), and bilateral renal agenesis or hypoplasia (sex ratio = 1.95, 95% CI = 1.38 to 2.76). Notably, each of these anomalies is also associated with childhood cancer risk (hazard ratio [HR] = 2.6, 95% CI = 2.1 to 3.2 for craniosynostosis; HR = 2.5, 95% CI = 1.7 to 3.7 for left ventricular outflow tract defects; and HR = 3.5, 95% CI = 2.3 to 5.2 for bilateral renal agenesis or hypoplasia) (2).

Although both sex and birth defect status have been individually evaluated as risk factors for childhood cancer, the extent to which the male excess in childhood cancer incidence may be attributable to the male excess in the prevalence of birth defects is unknown. As sex determination occurs at the moment of conception and onset of most major birth defects occurs during organogenesis in weeks 3–16 of gestation (10,11), birth defect status may be a mediator in the sex–childhood cancer relationship. Here we conduct a mediation analysis using a population-based study of over 10 000 000 live births to quantify the proportion of the association between male sex and childhood cancer that is mediated by birth defects.

## Methods

### Study Design

The GOBACK study was designed using population-based state registries to evaluate the association between birth defects and childhood cancer (2). This analysis uses the GOBACK data, which are described briefly below; further details are published elsewhere (2).

### Birth Certificate Data

The study included all recorded live births in Texas from 1999 to 2013, Arkansas from 1995 to 2011, Michigan from 1992 to 2011, and North Carolina from 2003 to 2012; differences in study years reflect data availability from state-specific registries (Supplementary Table 1, available online). Demographic and birth data were obtained from birth certificates.

**Table 1.** Infant and maternal characteristics of children included in this analysis

	Noncancer births, No. (%)			Childhood cancer births, No. (%)		
Category	Male	Female	Total	Male	Female	Total
Total	5 200 335 (51.2)	4 965 629 (48.8)	10 165 964 (100)	8044 (53.2)	7066 (46.8)	15 110 (100)
Any birth defect	319 480 (6.1)	217 963 (4.4)	537 442 (5.3)	1186 (14.7)	938 (13.3)	2,124 (14.1)
Chromosomal anomaly	11 013 (0.2)	10 915 (0.2)	21 928 (0.2)	166 (2.4)	172 (2.7)	338 (2.2)
Genetic anomaly	12 736 (0.3)	12 308 (0.3)	25 044 (0.2)	190 (2.7)	193 (3.1)	383 (2.5)
Nonsyndromic birth defect	306 744 (5.9)	205 655 (4.2)	512 399 (5.0)	996 (12.7)	745 (10.8)	1741 (11.5)
Maternal race/ethnicity						
Non-Hispanic white	3 314 406 (63.7)	3 152 974 (63.5)	6 467 380 (63.6)	5313 (66.1)	4,619 (65.4)	9932 (65.7)
Non-Hispanic black	778 429 (15.0)	752 815 (15.2)	1 531 244 (15.1)	890 (11.1)	827 (11.7)	1717 (11.4)
Other	1 107 500 (21.3)	1 059 840 (21.3)	2 167 340 (21.3)	1841 (22.8)	1,620 (22.9)	3461 (22.9)
Maternal education						
Less than high school	1 140 726 (21.9)	1 096 149 (22.1)	2 236 875 (22.0)	1714 (21.3)	1597 (22.6)	3311 (21.9)
High school	1 484 689 (28.6)	1 418 830 (28.6)	2 903 519 (28.6)	2369 (29.5)	2037 (28.8)	4406 (29.2)
Greater than high school	2 239 554 (6.5)	2 130 883 (42.9)	4 370 437 (43.0)	3500 (43.5)	3049 (43.2)	6549 (43.3)
Missing	335 366 (6.5)	319 767 (6.4)	655 133 (6.4)	461 (5.7)	383 (5.4)	844 (5.6)
Birth weight						
Low birth weight (<2500g)	387 058 (7.4)	427,651 (8.6)	814 709 (8.0)	614 (7.6)	591 (8.4)	1205 (8.0)
Normal birth weight (2500–3999 g)	4 129 384 (79.4)	4 077 298 (82.1)	8 206 682 (80.7)	6166 (76.7)	5645 (79.9)	11 811 (78.2)
High birth weight (≥4000 g)	500 295 (9.6)	285 463 (5.7)	785 758 (7.7)	908 (11.3)	512 (7.3)	1420 (9.4)
Missing	183 598 (3.5)	175 217 (3.5)	358 815 (3.5)	356 (4.4)	318 (4.5)	674 (4.5)
Gestational age						
<28 weeks	34 615 (0.7)	30 553 (0.6)	65 168 (0.6)	66 (0.8)	40 (0.6)	106 (0.7)
28 to 37 weeks	984 916 (18.9)	889 045 (17.9)	1 873 961 (18.4)	1625 (20.2)	1378 (19.5)	3003 (19.9)
≥38 weeks	4 148 337 (79.8)	4 016 498 (80.9)	8 164 835 (80.3)	6293 (78.2)	5603 (79.3)	11 896 (78.7)
Missing	32 467 (0.6)	29 533 (0.6)	62 000 (0.6)	60 (0.7)	45 (0.6)	105 (0.7)
Plurality						
Singleton	5 040 061 (96.9)	4 807 125 (96.8)	9 847 186 (96.9)	7800 (97.0)	6871 (97.3)	14 671 (97.1)
Multiple	159 353 (3.1)	157 685 (3.2)	317 038 (3.1)	244 (3.0)	193 (2.7)	437 (2.9)
Missing	921 (0.0)	819 (0.0)	1740 (0.0)	0 (0.0)	2 (0.0)	2 (0.0)
State						
Arkansas	321 638 (6.2)	306 411 (6.2)	628 049 (6.2)	562 (7.0)	475 (6.7)	1037 (6.9)
Michigan	1 314 886 (25.3)	1 251 418 (25.2)	2 566 304 (25.2)	2166 (26.9)	1933 (27.4)	4099 (27.1)
North Carolina	633 709 (12.2)	604 544 (12.2)	1 238 253 (12.2)	719 (8.9)	606 (8.6)	1325 (8.8)
Texas	2 930 102 (56.3)	2 803 256 (56.5)	5 733 358 (56.4)	4597 (57.2)	4052 (57.4)	8649 (57.2)

### Birth Defects Ascertainment

Birth defects surveillance systems in Texas, Arkansas, and North Carolina employ active ascertainment methods; passive ascertainment methods are used in Michigan (6,12–16). Specific birth defects included were “major” defects (14,15) included as part of the National Birth Defects Prevention Network’s annual surveillance report (12) or the National Birth Defects Prevention Study case definitions (14).

### Childhood Cancer Ascertainment

Data on cancer site, morphology, behavior, and age at diagnosis were obtained from the population-based cancer registries of each state. All participating cancer registries follow the standards of the National Program of Cancer Registries and are certified by the North American Association of Central Cancer Registries (17).

The childhood cancer cases were coded into groups according to the International Classification of Childhood Cancer, Third Edition. Children diagnosed younger than age 18 years are included. In the subset of children with more than 1 cancer diagnosis (n = 235), we included only the first primary cancer.

### Record Linkage

Within each state, birth defects and cancer registries were linked to birth certificates. Individual records in the assembled birth cohort were linked across data sources using deterministic and probabilistic linkage. Over 95% of birth defect cases and over 70% of childhood cancer cases across the cohort were matched to birth certificates (6,16,18). Linked data were deidentified and systematically cleaned, harmonized, and coded across states.

### Statistical Analysis

We conducted counterfactual mediation analysis (19) to estimate the direct and indirect effects of sex on risk of childhood cancer, modeling sex as the exposure (male or female), birth defect status as the mediator (any or none), and cancer type as the outcome (cancer overall and by subtype). The counterfactual mediation analysis provides a framework whereby the total effect of an exposure on an outcome can be decomposed into a natural direct effect and natural indirect effect. In this analysis, the natural direct effect is defined as NDE_a, a*_(a*) = *E*[T_aMa*_]/*E*[T_a*Ma*_] and the natural indirect effect is defined as NIE_a, a*_(*a*) = *E*[*T_aMa_*]/*E*[T_aMa*_], where *T_a_* and *M_a_* denote the values of the time-to-event cancer outcome and birth defect mediator that would have been observed if the exposure *A* had been set to level *a*. *T_am_* is the value of the time-to-event cancer outcome that would have been observed if the exposure *A* and mediator *M* had been set to levels *a* and *m*, respectively. We have included a directed acyclic graph of our model in Supplementary Figure 1 (available online). The natural direct effect captures the influence of infant sex on childhood cancer risk if the link between infant sex and the mediator (birth defect status) was prevented or removed hypothetically. This simulates a scenario wherein the sample distributions of the mediator are no longer dependent on infant sex. By contrast, the natural indirect effect captures the effect of infant sex on childhood cancer risk that operates through birth defects status. Consistent with previous analyses (12,20), we assessed the effect of sex on birth defects status using logistic regression models and the effect of both sex and birth defects status on childhood cancer risk using Cox proportional hazards models. Person-years were calculated as time from birth to death, cancer diagnosis, or end of study period (December 31, 2011, in Arkansas and Michigan; December 31, 2012, in North Carolina; and December 31, 2013, in Texas). We estimated standard errors of the hazard ratios through the delta method. The proportion mediated was reported only if the hazard ratios for the direct and indirect effects were in the same direction (19,21).

**Figure 1.**
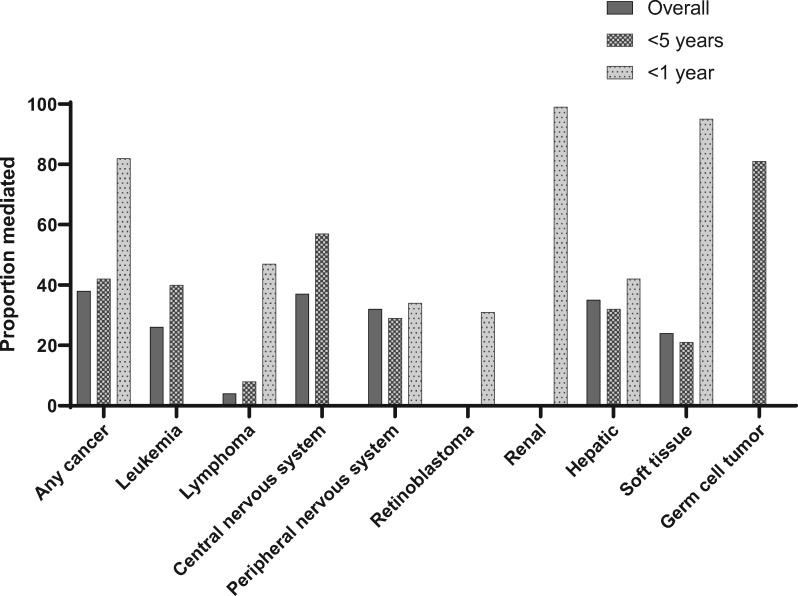
Proportion of the sex–cancer association mediated by birth defects status for major tumor types, by age at diagnosis.

Counterfactual mediation analyses assume that, conditional on the covariates, there is no confounding of 1) the exposure-outcome relationship, 2) the mediator-outcome relationship, and 3) the exposure-mediator relationship, and that 4) there is no effect of the exposure that itself confounds the mediator-outcome relationship. Assumptions 1 and 3 are likely to hold when the main exposure variable is infant sex. Birth year, state, maternal race and ethnicity (non-Hispanic White, non-Hispanic Black, other), maternal education (less than high school, high school, more than high school), maternal age (continuous), and plurality (singleton vs multiple) were identified a priori as potential mediator-outcome confounders to address assumption 2. Finally, there are no known effects of infant sex that may confound the relationship of birth defect and childhood cancer. Therefore we do not believe that assumption 4 is violated.

Because of lower success rates of linkage to birth records among adolescent cancer cases, we conducted a subgroup analysis limited to cancers diagnosed at age younger than 5 years. Additionally, due to observations that the association between birth defects and cancer is strongest for those diagnosed with cancer at the youngest ages (3,7), we conducted analyses restricted to children diagnosed with cancer before 1 year of age. We conducted analyses for only those cancers with at least 5 cases diagnosed among each age group. Because of the documented association between certain childhood cancers and chromosomal anomalies or single gene disorders, which are generally independent of sex (9,22–24), we conducted subgroup analyses excluding children with chromosomal anomalies (n = 22 420), or single gene disorders (neurofibromatosis type I and tuberous sclerosis) (n = 2972). This was done to quantify the mediation effect of nonsyndromic birth defects alone. Finally, to account for multiple comparisons, we corrected the *P* values for the natural indirect effects using the Benjamini-Hochberg method of the false discovery rate, setting α = 0.05. Statistical analyses were performed in SAS 9.4. All statistical tests were 2-sided.

## Results

Our dataset included 10 181 074 children (5 208 379 male; 4 972 695 female), including 15 110 with cancer diagnoses (8044 male; 7066 female), 539 567 children with birth defects diagnoses (320 666 male; 218 901 female), and 2124 co-occurring cases (children with both cancer and one or more birth defect diagnoses: 1186 male; 938 female). Table 1 shows demographic characteristics of the study population. Males were more likely to be diagnosed with any childhood cancer (HR = 1.09, 95% CI = 1.05 to 1.12) and were more likely to have a birth defect (odds ratio = 1.42, 95% CI = 1.41 to 1.43). The associations between sex and childhood cancer are presented in Supplementary Table 2 (available online), and those between sex and birth defects status are presented in Supplementary Table 3 (available online).

**Table 2.** Hazard ratios (HRs) from the mediation analysis for the association between sex and childhood cancer mediated by birth defects status^a^

Cancer	Natural indirect effectHR (95% CI)	Natural direct effectHR (95% CI)	TotalHR (95% CI)	Proportion mediated, %^b^	FDR-adjusted *P* value for the natural indirect effect
Any cancer	1.03 (1.02 to 1.03)	1.05 (1.02 to 1.09)	1.08 (1.05 to 1.12)	38	<.001
Leukemia	1.03 (1.02 to 1.03)	1.09 (1.03 to 1.16)	1.12 (1.06 to 1.19)	26	<.001
Acute lymphoblastic leukemia	1.02 (1.01 to 1.02)	1.14 (1.07 to 1.22)	1.16 (1.08 to 1.24)	11	<.001
Acute myeloid leukemia	1.07 (1.06 to 1.08)	1.01 (0.86 to 1.18)	1.08 (0.92 to 1.26)	93	<.001
Other leukemia	1.08 (1.06 to 1.09)	0.88 (0.73 to 1.06)	0.95 (0.79 to 1.14)	−	<.001
Lymphoma	1.02 (1.01 to 1.02)	1.52 (1.36 to 1.70)	1.54 (1.38 to 1.72)	4	<.001
Hodgkin lymphoma	1.00 (0.99 to 1.00)	1.50 (1.21 to 1.86)	1.49 (1.20 to 1.85)	−	.52
Non-Hodgkin lymphoma	1.02 (1.01 to 1.03)	1.44 (1.20 to 1.74)	1.47 (1.22 to 1.77)	6	<.001
Other lymphoma	1.02 (1.01 to 1.03)	1.61 (1.35 to 1.92)	1.65 (1.39 to 1.96)	6	<.001
Central nervous ystem	1.03 (1.02 to 1.03)	1.05 (0.98 to 1.12)	1.08 (1.01 to 1.16)	37	<.001
Ependymoma	1.01 (1.00 to 1.03)	1.33 (1.04 to 1.69)	1.35 (1.06 to 1.71)	6	.16
Medulloblastoma	1.03 (1.02 to 1.05)	1.59 (1.30 to 1.94)	1.64 (1.35 to 2.00)	8	<.001
Astrocytoma	1.03 (1.03 to 1.04)	0.95 (0.86 to 1.06)	0.98 (0.88 to 1.10)	−	<.001
Primitive neuroectodermal tumor	1.02 (1.00 to 1.04)	0.86 (0.59 to 1.26)	0.88 (0.60 to 1.29)	−	.04
Other central nervous system	1.03 (1.02 to 1.03)	1.00 (0.90 to 1.12)	1.03 (0.93 to 1.15)	84	<.001
Peripheral nervous system	1.04 (1.03 to 1.05)	1.10 (0.98 to 1.22)	1.14 (1.02 to 1.27)	32	<.001
Neuroblastoma	1.04 (1.03 to 1.05)	1.08 (0.97 to 1.21)	1.12 (1.01 to 1.25)	35	<.001
Other peripheral nervous system	1.09 (1.00 to 1.20)	5.79 (1.30 to 25.7)	6.32 (1.43 to 28.0)	10	.06
Retinoblastoma	1.02 (1.01 to 1.03)	0.98 (0.83 to 1.17)	1.01 (0.85 to 1.20)	−	<.001
Renal	1.03 (1.02 to 1.04)	0.78 (0.69 to 0.89)	0.81 (0.71 to 0.92)	−	<.001
Nephroblastoma	1.03 (1.02 to 1.04)	0.76 (0.67 to 0.88)	0.79 (0.69 to 0.90)	−	<.001
Other renal	1.04 (1.01 to 1.06)	0.99 (0.64 to 1.51)	1.02 (0.67 to 1.56)	−	.01
Hepatic	1.11 (1.09 to 1.13)	1.27 (1.00 to 1.60)	1.41 (1.11 to 1.78)	35	<.001
Hepatoblastoma	1.11 (1.09 to 1.13)	1.30 (1.01 to 1.67)	1.44 (1.12 to 1.85)	33	<.001
Other hepatic	1.14 (1.07 to 1.22)	1.02 (0.50 to 2.08)	1.17 (0.58 to 2.37)	86	<.001
Bone	1.01 (1.00 to 1.02)	1.00 (0.81 to 1.23)	1.00 (0.81 to 1.23)	−	.99
Osteosarcoma	1.00 (0.99 to 1.01)	0.99 (0.74 to 1.33)	1.00 (0.74 to 1.34)	−	.99
Ewing sarcoma	1.00 (0.99 to 1.01)	1.03 (0.72 to 1.47)	1.03 (0.72 to 1.47)	−	.90
Other bone	1.04 (1.00 to 1.07)	0.92 (0.55 to 1.56)	0.95 (0.57 to 1.61)	−	.06
Soft tissue	1.03 (1.02 to 1.03)	1.10 (0.98 to 1.24)	1.13 (1.01 to 1.27)	24	<.001
Any rhabdomyosarcoma	1.01 (1.00 to 1.02)	1.28 (1.06 to 1.56)	1.30 (1.07 to 1.57)	5	.02
Other rhabdomyosarcoma	1.02 (1.00 to 1.04)	1.34 (0.89 to 2.02)	1.37 (0.91 to 2.06)	6	.10
Alveolar rhabdomyosarcoma	1.01 (0.99 to 1.03)	1.01 (0.66 to 1.56)	1.02 (0.67 to 1.57)	39	.39
Embryonal rhabdomyosarcoma	1.01 (1.00 to 1.02)	1.37 (1.06 to 1.77)	1.38 (1.07 to 1.79)	3	.15
Other soft tissue	1.04 (1.03 to 1.05)	1.00 (0.87 to 1.16)	1.04 (0.90 to 1.21)	91	<.001
Germ cell tumor	1.07 (1.05 to 1.08)	0.83 (0.70 to 0.99)	0.89 (0.74 to 1.06)	−	<.001
Extracranial germ cell tumor	1.14 (1.11 to 1.18)	0.45 (0.33 to 0.62)	0.52 (0.37 to 0.71)	−	<.001
Gonadal germ cell tumor	1.02 (1.01 to 1.04)	1.15 (0.88 to 1.49)	1.17 (0.90 to 1.52)	16	.002
Intracranial germ cell tumor	1.05 (1.02 to 1.08)	1.03 (0.70 to 1.50)	1.08 (0.74 to 1.57)	64	<.001
Epithelial	1.01 (1.00 to 1.02)	0.54 (0.45 to 0.64)	0.55 (0.46 to 0.65)	−	.03
Any other	1.02 (1.00 to 1.05)	1.18 (0.80 to 1.73)	1.20 (0.82 to 1.77)	13	.05

a Analyses are adjusted for birth year, state, maternal race/ethnicity, maternal education, maternal age, and plurality. FDR = false discovery rate.

b Empty cells are present when the proportion mediated is not estimable because the natural direct and indirect effects are in opposite directions.

**Table 3.** Hazard ratios (HRs) from the mediation analysis for the association between sex and childhood cancer mediated by birth defect status; restricted to children younger than 1 year^a^

Cancer	Natural indirect effectHR (95% CI)^b^	Natural direct effectHR (95% CI)^b^	TotalHR (95% CI)^b^	Proportion mediated, %^c^	FDR-adjusted *P* value for the natural indirect effect
Any cancer	1.08 (1.07 to 1.09)	1.02 (0.95 to 1.09)	1.10 (1.02 to 1.18)	82	<.001
Leukemia	1.08 (1.07 to 1.09)	0.88 (0.75 to 1.02)	0.95 (0.81 to 1.11)	−	<.001
Acute lymphoblastic leukemia	1.03 (1.02 to 1.05)	0.97 (0.77 to 1.23)	1.00 (0.79 to 1.27)	−	<.001
Acute myeloid leukemia	1.12 (1.09 to 1.15)	0.70 (0.53 to 0.92)	0.78 (0.59 to 1.03)	−	<.001
Other leukemia	1.13 (1.10 to 1.16)	0.98 (0.72 to 1.33)	1.10 (0.81 to 1.49)	−	<.001
Lymphoma	1.07 (1.04 to 1.10)	1.08 (0.76 to 1.55)	1.16 (0.81 to 1.66)	47	<.001
Hodgkin lymphoma	−	−	−	−	
Non-Hodgkin lymphoma	−	−	−	−	
Other lymphoma	1.07 (1.04 to 1.10)	1.11 (0.75 to 1.65)	1.19 (0.80 to 1.77)	42	<.001
Central nervous system	1.09 (1.07 to 1.11)	0.99 (0.83 to 1.20)	1.09 (0.91 to 1.30)	−	<.001
Ependymoma	1.06 (1.02 to 1.10)	1.17 (0.64 to 2.13)	1.24 (0.68 to 2.25)	28	.01
Medulloblastoma	1.12 (1.07 to 1.18)	1.18 (0.66 to 2.10)	1.32 (0.74 to 2.35)	44	<.001
Astrocytoma	1.08 (1.05 to 1.11)	0.95 (0.69 to 1.31)	1.02 (0.74 to 1.41)	−	<.001
Primitive neuroectodermal tumor	−	−	−	−	
Other central nervous system	1.10 (1.07 to 1.13)	0.98 (0.75 to 1.28)	1.08 (0.83 to 1.41)	−	<.001
Peripheral nervous system	1.07 (1.06 to 1.08)	1.16 (0.99 to 1.35)	1.24 (1.06 to 1.45)	34	<.001
Neuroblastoma	1.07 (1.06 to 1.08)	1.15 (0.98 to 1.34)	1.23 (1.05 to 1.44)	35	<.001
Other peripheral nervous system	−	−	−	−	
Retinoblastoma	1.04 (1.03 to 1.06)	1.10 (0.87 to 1.40)	1.15 (0.91 to 1.46)	31	<.001
Renal	1.07 (1.05 to 1.09)	1.00 (0.77 to 1.31)	1.07 (0.82 to 1.39)	99	<.001
Nephroblastoma	1.07 (1.05 to 1.10)	0.95 (0.72 to 1.27)	1.02 (0.77 to 1.36)	−	<.001
Other renal	−	−	−	−	
Hepatic	1.15 (1.11 to 1.18)	1.27 (0.90 to 1.80)	1.46 (1.03 to 2.07)	42	<.001
Hepatoblastoma	1.14 (1.11 to 1.18)	1.26 (0.88 to 1.79)	1.43 (1.00 to 2.04)	42	<.001
Other hepatic	−	−	−	−	
Bone	−	−	−	−	
Osteosarcoma	−	−	−	−	
Ewing sarcoma	−	−	−	−	
Other bone	−	−	−	−	
Soft tissue	1.07 (1.05 to 1.09)	1.00 (0.78 to 1.30)	1.08 (0.83 to 1.39)	95	<.001
Any rhabdomyosarcoma	1.03 (1.00 to 1.06)	0.87 (0.54 to 1.39)	0.89 (0.56 to 1.43)	−	.04
Other rhabdomyosarcoma	−	−	−	−	
Alveolar rhabdomyosarcoma	−	−	−	−	
Embryonal rhabdomyosarcoma	1.04 (1.00 to 1.09)	0.59 (0.31 to 1.13)	0.62 (0.32 to 1.18)	−	.05
Other soft tissue	1.10 (1.07 to 1.12)	1.07 (0.79 to 1.45)	1.17 (0.86 to 1.59)	60	<.001
Germ cell tumor	1.13 (1.10 to 1.16)	0.95 (0.72 to 1.26)	1.08 (0.82 to 1.42)	−	<.001
Extracranial germ cell tumor	1.18 (1.14 to 1.22)	0.38 (0.26 to 0.55)	0.44 (0.30 to 0.65)	−	<.001
Gonadal germ cell tumor	1.05 (1.01 to 1.08)	17.9 (5.61 to 57.2)	18.8 (5.88 to 59.8)	5	.01
Intracranial germ cell tumor	1.13 (1.05 to 1.22)	0.91 (0.39 to 2.16)	1.03 (0.44 to 2.43)	−	.002
Epithelial	−	−	−	−	
Any other	1.05 (1.00 to 1.10)	1.03 (0.50 to 2.12)	1.08 (0.53 to 2.22)	62	.06

a Analyses are adjusted for birth year, state, maternal race/ethnicity, maternal education, maternal age, and plurality. FDR = false discovery rate.

b Empty cells are present when there were fewer than 5 cancer cases in the category.

c Empty cells are present when the proportion mediated is not estimable because the natural direct and indirect effects are in opposite directions.

Figure 1 shows the proportion mediated for all cancers combined and by major diagnostic category, for all children and within subgroups by age at diagnosis. Among cancer categories where we could compute a proportion mediated for more than one age group, we generally observed that the proportion mediated increased with decreasing age. We observed statistically significant mediation of the association between sex and childhood cancer by birth defect status among children younger than age 18 years (proportion mediated [PM] = 38%; Table 2). In analyses of specific cancer types, we observed variation of the estimated proportion mediated among several cancers, including acute myeloid leukemia (PM = 93%), neuroblastoma (PM = 35%), hepatoblastoma (PM = 33%), non-Hodgkin lymphoma (PM = 6%), and soft tissue sarcomas (PM = 24%). The indirect effects for most cancers were statistically significant, albeit with modest effect sizes (<1.05). The largest indirect effects we observed were for hepatoblastoma (HR_NIE_ = 1.11, 95% CI = 1.09 to 1.13) and extracranial and extragonadal germ cell tumors (HR_NIE_ = 1.14, 95% CI = 1.11 to 1.18).

**Figure 2.**
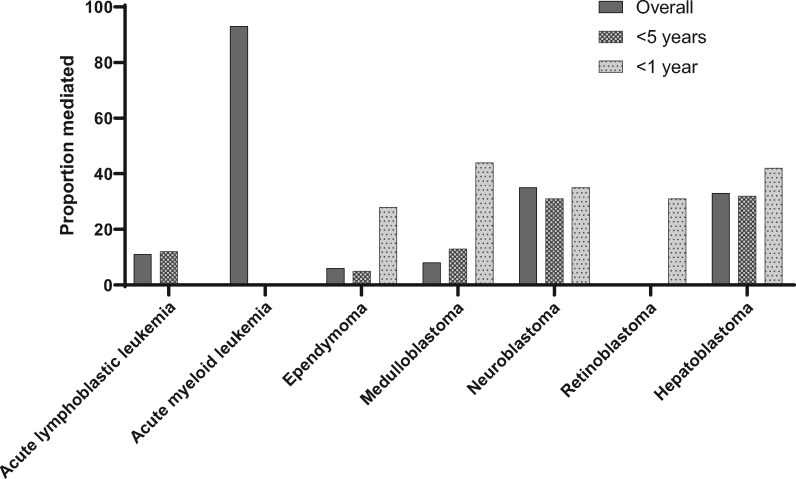
Proportion of the sex and cancer association mediated by birth defects status for selected leukemias, central nervous system tumors, and embryonal tumors, by age at diagnosis.

In analyses of children younger than 5 years at cancer diagnosis, we observed similar results (Supplementary Table 4, available online). The proportion of the effect of sex on cancer risk among children younger than 5 years mediated by birth defects status was 42%. In analyses of children younger than 1 year of age at cancer diagnosis, we observed stronger indirect effects, and the proportion of the sex and childhood cancer association mediated by birth defects status among infants was 82% (Table 3). The proportion mediated in infants was moderate to high in nearly every cancer type where this statistic could be calculated, including ependymoma (28%), medulloblastoma (44%), neuroblastoma (35%), retinoblastoma (31%), hepatoblastoma (42%), and non-rhabdomyosarcoma soft tissue sarcomas (60%); the only exception was gonadal germ cell tumors (5%). Figure 2 shows cancer-specific results for selected leukemias, central nervous system tumors, and embryonal tumors.

In analyses restricted to nonsyndromic birth defects (Supplementary Table 5, available online), we observed a weakened indirect effect for acute lymphoblastic leukemia and acute myeloid leukemia (PM = 4% and 29%, respectively). However, associations for solid tumors remained largely consistent with those observed when considering all anomaly types (chromosomal, single gene, and nonsyndromic).

## Discussion

In this population-based analysis of over 10 000 000 live births, we observed that birth defects status is likely to explain a substantial proportion of the sex ratio disparity in childhood cancer. The proportion mediated varied considerably by cancer type and age at diagnosis; notably, we estimate that 82% of the male excess in childhood cancer incidence among infants is mediated by birth defect status.

The increased risk of cancer among male adults compared with female adults is well established (25), and differences in risk are at least partially attributable to differences in risk behaviors such as alcohol and tobacco use (26,27). By contrast, there is a paucity of published data on the possible causal pathways underlying the sex disparity in childhood cancer incidence. In one study (28), the authors conducted a mediation analysis to examine whether the sex and childhood cancer relationship is mediated by birth weight. They reported modest mediation for all cancers combined and for acute lymphoblastic leukemia. However, birth weight did not explain a large proportion of the sex-cancer relationships examined.

We observed strong mediation effects for the embryonal tumors neuroblastoma and hepatoblastoma. Notably, these tumors were the 2 most commonly associated with nonchromosomal birth defects in our recent assessment. We also observed nearly complete mediation of the sex association with acute myeloid leukemia among children of all ages (PM = 93%). Birth defects did not mediate a large proportion of the sex effect on medulloblastoma for all children or children diagnosed at younger than 5 years old, but we did observe a larger mediation effect among infants (PM = 44%).

We observed a wide range of proportion mediated. Each childhood cancer subtype has different associations with child sex, thus the sex-specific incidence of each cancer type is one factor driving these results. Additionally, GOBACK data showed that birth defects are more strongly associated with some childhood cancer types than others (2). Thus, cancers that are strongly associated with birth defects, such as hepatoblastoma, are more likely to have a strong mediation effect.

For some cancers, we observed age-dependent variation in our results when comparing analyses of the entire study population with subgroup analyses among children younger than 5 years and younger than 1 year of age. It is well established that the sex ratio among childhood cancer cases differs by age (1). Analyses of the birth defect and cancer associations have also seen age-dependent results, with stronger effect estimates for younger age at cancer onset (3,7). Finally, evidence suggests that there are age-dependent biologically distinct subtypes of a single cancer (ie, acute lymphoblastic leukemia) based on the differing etiology and molecular subtypes that vary by age at diagnosis (29,30). We believe that these factors are driving the differences that we observed by age at diagnosis.

For some cancer types, we observed positive, statistically significant, natural indirect effects with natural direct effects below the null. These results indicate that the pathway through birth defects is driving up the male incidence for that particular cancer type, although male sex has an inverse association with that particular cancer through all other pathways. For example, there was a strong natural indirect effect for extracranial germ cell tumors (HR_NIE_ = 1.14, 95% CI = 1.11 to 1.18), whereas the natural direct effect showed a strong inverse association (HR_NDE_ = 0.45, 95% CI = 0.33 to 0.62). These results indicate that, in the absence of an effect of birth defects, the female excess in extracranial germ cell tumor incidence would be even more pronounced than currently observed. We observed similar patterns for acute myeloid leukemia among children younger than 5 years and younger than 1 year of age at diagnosis.

When we conducted analyses of nonsyndromic birth defects only, we observed a decrease in the proportion mediated for acute lymphoblastic leukemia and acute myeloid leukemia, whereas most other results remained unchanged (Supplementary Table 5, available online). Risk of both acute lymphoblastic leukemia and acute myeloid leukemia are increased among children with Down syndrome (23,31,32). Furthermore, results from our data (Supplementary Table 3, available online) and other analyses have shown a male excess among infants born with Down syndrome (9,33). Thus the exclusion of Down syndrome in this subgroup analysis is likely driving these results.

There are limitations to consider when interpreting these results. Because of linkage procedures, children who migrated away from their birth state would be lost to follow-up, therefore would not be identified if they subsequently developed cancer. Our linkage success rates among children age 0-5 years, 6-10 years, and 11 years and over at cancer diagnosis were 74%, 66%, and 60%, respectively. These rates are similar to those observed in previous studies (18,30) and were not differential by child’s sex in any age group. Additionally, there is evidence that suggests out-of-state migration is nondifferential according to birth defect status (34), which limits the possibility of differential misclassification. We observed unexpected sex ratios of some tumor types (Supplementary Table 2, available online), most notably osteosarcoma and Ewing sarcoma. The unexpected sex ratios are likely because of the lower linkage success rates of older cancer cases. In osteosarcoma and Ewing sarcoma occurring at younger than 18 years of age, the male excess is due almost entirely to adolescent cases; there is nearly no difference in sex ratio among younger cases (1). Despite this limitation, we do not expect that early life migration is differential by birth defects status or child sex, as noted above. Therefore it is unlikely that results were influenced by lower linkage success of older cases. There may be limitations in birth defect ascertainment if the presence of cancer caused the appearance of a birth defect that was in fact a structural displacement due to cancerous growth. However, we have previously shown that birth defect-cancer associations for which this is a concern (ie, hydrocephaly secondary to central nervous system tumors) remained statistically significant after exclusion of cases with these combinations diagnosed in infancy (2). Because of the small sample size of minority populations in this dataset (Table 1) (2), we categorized Hispanic, Asian, American Indian/Alaskan Native, and other or unknown mothers into the “Other” racial and ethnic category. Finally, although there are some factors such as birth weight that are associated with infant sex, the sex determination of the fetus nearly always precedes these factors and therefore they would not confound the sex and birth defect relationship or the sex-cancer relationship. However, it is possible that unmeasured confounders exist for the mediator-outcome relationship. The underlying causes of the strong association between birth defects and childhood cancer risk are unknown and may be because of shared environmental risk factors, unidentified developmental disorders, or genetic syndromes.

In conclusion, we evaluated mediation of the sex and childhood cancer relationship by birth defects using a population-based study design with a very large sample size. Our results suggest that birth defects mediate a substantial proportion of the overall relationship between sex and childhood cancer, particularly among younger children. Although approximately 60% of the male excess in childhood cancer incidence among children age younger than 18 years remains unexplained, these findings add to our understanding of the causal pathway of male sex as a risk factor for childhood cancer. These results may assist in refinement of risk stratifications and surveillance strategies among children with birth defects as we develop an increased understanding of the pathways involved in carcinogenesis in this population. Other possible mechanisms underlying the male excess include sex-specific genetic factors or immune response (35–37). Additional studies are under way to characterize the biology underlying these observations.

## Funding

This work was supported by the Cancer Prevention & Research Institute of Texas (CPRIT RP140258, RP170071, RP160097, and RP160283), National Cancer Institute at the National Institutes of Health (CA125123), Arkansas Biosciences Institute, Alex’s Lemonade Stand Foundation, and the Children’s Cancer Research Fund.

## Notes

**Role of the funder:** The funders had no role in the design of the study; the collection, analysis, and interpretation of the data; the writing of the manuscript; and the decision to submit the manuscript for publication.

**Disclosures:** The authors have no conflicts of interest to disclose.

**Role of the authors**: ELM and PJL had full access to all the data in the study and take responsibility for the integrity of the data and the accuracy of the data analysis. ELM and PJL: Concept and design. ELM, PJL, JMS, TAD, WNN, PHL, MAC, REM, and SEP: Acquisition, analysis, or interpretation of data. ELM, PJL, and JMS: Drafting of the manuscript. All authors: Critical revision of the manuscript for important intellectual content. ELM: Statistical analysis. PJL: Obtained funding. ELM, PJL, JMS, TAD, WNN, MAC, REM, and SEP: Administrative, technical, or material support. ELM and PJL: Supervision.

**Acknowledgments:** We acknowledge the various state agencies that made this work possible, including the Texas Birth Defects Registry, Texas Cancer Registry, Texas Center for Health Statistics, Texas Department of State Health Services, Arkansas Reproductive Health Monitoring System, Arkansas Cancer Registry, Michigan Department of Health and Human Services, North Carolina Central Cancer Registry, North Carolina Birth Defects Monitoring Program, North Carolina State Center for Health Statistics, and the North Carolina Division of Public Health.

## Data availability statement

The data underlying this article cannot be shared publicly due to state restrictions and privacy laws. The data will be shared on reasonable request to the corresponding author, with appropriate approvals by each state's institutional review board.

## Supplementary Material

pkaa052_Supplementary_Data
