# Supplementary material for: Male Sex and the Risk of Childhood Cancer: The Mediating Effect of Birth Defects
Source: JNCI Cancer Spectr. 2020 Jun 11;4(5):pkaa052. doi: 10.1093/jncics/pkaa052 (PMC7583156; doi:10.1093/jncics/pkaa052)

Supplementary Table 1. Distriubtion of births, birth defects, and cancers by state

	Texas	Arkansas	Michigan	North Carolina	Total
Birth years	1999-2013	1995-2011	1992-2011	2003-2012	1992-2013
Births, *No.* (%)[a]	5,742,007 (56.4)	629,086 (6.2)	2,570,403 (25.2)	1,239,578 (12.2)	10,181,074
Birth defects, *No.* (%)[b]	251,516 (4.4)	23,341 (3.7)	224,026 (8.7)	40,684 (3.3)	539,567
Age cutoff for birth defects diagnoses	1 year	2 years	2 years	1 year	
Cancers, *No.* (%)[c]	8,649 (0.2)	1,037 (0.2)	4,099 (0.2)	1,325 (0.1)	15,110
Co-occurring cases[d], *No.*	918	75	1,012	119	2,124

[a]Percentage of total births in the assembled study cohort.

[b]Number and percentage of children in the cohort diagnosed with any chromosomal anomaly, single gene disorder, or non-syndromic birth defect.

[c]Percentage of total births within state.

[d]Co-occurring birth defect and cancer.

Supplementary Table 2. Associations between sex and childhood cancer

	male	female	HR (95% CI)
**non-cancer births**	5200335	4965629	
**Any cancer**	8044	7066	1.09 (1.05 to 1.12)
**Leukemia**	2492	2116	1.13 (1.06 to 1.19)
Acute lymphoblastic leukemia	1930	1589	1.16 (1.09 to 1.24)
Acute myeloid leukemia	328	290	1.08 (0.92 to 1.27)
Other leukemia	234	237	0.95 (0.79 to 1.13)
**Lymphoma**	842	519	1.55 (1.39 to 1.73)
Hodgkin lymphoma	212	135	1.50 (1.21 to 1.86)
Non-Hodgkin lymphoma	282	183	1.47 (1.22 to 1.77)
Other lymphoma	348	201	1.65 (1.39 to 1.97)
**Central Nervous System**	1851	1632	1.08 (1.01 to 1.16)
Ependymoma	161	114	1.35 (1.06 to 1.71)
Medulloblastoma	264	153	1.65 (1.35 to 2.01)
Astrocytoma	673	653	0.99 (0.89 to 1.10)
Primitive neuroectodermal tumor	52	56	0.89 (0.61 to 1.29)
Other central nervous system	716	661	1.03 (0.93 to 1.15)
**Peripheral Nervous System**	708	589	1.15 (1.03 to 1.28)
Neuroblastoma	695	587	1.13 (1.01 to 1.26)
Other peripheral nervous system	13	2	6.20 (1.40 to 27.4)
**Retinoblastoma**	263	249	1.01 (0.85 to 1.20)
**Renal**	426	502	0.81 (0.71 to 0.92)
Nephroblastoma	382	461	0.79 (0.69 to 0.91)
Other renal	44	41	1.03 (0.67 to 1.57)
**Hepatic**	173	117	1.41 (1.12 to 1.79)
Hepatoblastoma	156	103	1.45 (1.13 to 1.86)
Other hepatic	17	14	1.16 (0.57 to 2.35)
**Bone**	183	174	1.00 (0.82 to 1.24)
Osteosarcoma	91	87	1.00 (0.74 to 1.34)
Ewing sarcoma	64	59	1.04 (0.73 to 1.47)
Other bone	28	28	0.96 (0.57 to 1.61)
**Soft tissue**	612	513	1.14 (1.01 to 1.28)
Any rhabdomyosarcoma	241	177	1.30 (1.07 to 1.58)
Other rhabdomyosarcoma	56	39	1.37 (0.91 to 2.06)
Alveolar rhabdomyosarcoma	43	40	1.03 (0.67 to 1.58)
Embryonal rhabdomyosarcoma	142	98	1.38 (1.07 to 1.79)
Other soft tissue	371	336	1.05 (0.91 to 1.22)
**Germ cell tumor**	240	257	0.89 (0.75 to 1.06)
Extracranial germ cell tumor	57	105	0.52 (0.38 to 0.72)
Gonadal germ cell tumor	126	102	1.18 (0.91 to 1.53)
Intracranial germ cell tumor	57	50	1.09 (0.74 to 1.59)
**Epithelial**	199	346	0.55 (0.46 to 0.65)
**Any other**	58	46	1.20 (0.82 to 1.77)

Supplementary Table 3. Associations between sex and birth defects

	male	female	OR (95% CI)
**non birth defect births**	4887713	4753794	
**Any birth defect**	320666	218901	1.42 (1.41 to 1.43)
**Any chromosomal anomaly**	11179	11087	0.98 (0.96 to 1.01)
**Any genetic anomaly**	12926	12501	1.01 (0.98 to 1.03)
**Chromosomal anomalies and single gene disorders**			
Trisomy 13	462	445	1.01 (0.89 to 1.15)
Trisomy 18	555	911	0.59 (0.53 to 0.66)
Trisomy 21	6971	6151	1.10 (1.07 to 1.14)
Gonadal dysgenesis	46	830	0.05 (0.04 to 0.07)
del 13q	24	23	1.02 (0.57 to 1.80)
del 22q	162	162	0.97 (0.78 to 1.21)
**Single gene anomaly**	1973	1593	1.21 (1.13 to 1.29)
Neurofibromatosis	270	237	1.11 (0.93 to 1.32)
Tuberous sclerosis	1707	1357	1.22 (1.14 to 1.31)
**Non-syndromic birth defects**			
**Congenital anomalies of the nervous system**	22245	18997	1.14 (1.12 to 1.16)
Anencephalus	446	511	0.85 (0.75 to 0.96)
Spina bifida without anencephaly	1689	1807	0.91 (0.85 to 0.97)
Hydrocephaly without spina bifida	5082	3794	1.30 (1.25 to 1.36)
Encephalocele	431	479	0.88 (0.77 to 1.00)
Microcephaly	4772	5612	0.83 (0.80 to 0.86)
Holoprosencephaly	1997	1942	1.00 (0.94 to 1.06)
Other CNS cong anomalies	12106	8991	1.31 (1.27 to 1.35)
**Congenital anomalies of the eye**	15052	14020	1.04 (1.02 to 1.07)
Anopthalmia or micropthalmia	1253	1340	0.91 (0.84 to 0.98)
Aniridia	76	86	0.86 (0.63 to 1.17)
Congenital cataract	1120	1149	0.95 (0.87 to 1.03)
Other eye anomalies	13689	12601	1.06 (1.03 to 1.08)
**Congenital anomalies of the respiratory system**	41862	36795	1.11 (1.09 to 1.12)
Choanal atresia	739	716	1.00 (0.91 to 1.11)
Lung agenesis or hypoplasia	2278	1709	1.30 (1.22 to 1.38)
Other respiratory anomalies	40079	35409	1.10 (1.09 to 1.12)
**Congenital anomalies of the heart and circulatory system**	81442	76271	1.04 (1.03 to 1.05)
Pulmonary artery anomalies	10628	10034	1.03 (1.00 to 1.06)
Single ventricle	566	426	1.29 (1.14 to 1.47)
Tricuspid valve atresia and stenosis	820	757	1.05 (0.95 to 1.16)
Left ventricular outflow tract defects	4779	3294	1.41 (1.35 to 1.48)
Aortic valve stenosis	1629	957	1.66 (1.53 to 1.79)
Coarctation of aorta	3135	2299	1.33 (1.26 to 1.40)
Hypoplastic left heart syndrome	2094	1497	1.36 (1.27 to 1.45)
Interrupted aortic arch type a or c	348	321	1.05 (0.91 to 1.23)
Right ventricular outflow tract defects	5160	5914	0.85 (0.82 to 0.88)

Ebstein anomaly	456	442	1.00 (0.88 to 1.14)
Pulmonary valve atresia and stenosis	4814	5584	0.84 (0.81 to 0.87)
Conotruncal defects	5654	4025	1.37 (1.31 to 1.42)
Common truncus	470	453	1.01 (0.89 to 1.15)
Transposition of great vessels	3184	1903	1.63 (1.54 to 1.72)
Tetralogy of Fallot	2180	1782	1.19 (1.12 to 1.27)
Septal defects	60006	58743	0.99 (0.98 to 1.01)
Atrial septal defect	48864	46308	1.03 (1.01 to 1.04)
Ventricular septal defect	25367	28962	0.85 (0.84 to 0.87)
Endocardial cushion defect	2312	2576	0.87 (0.83 to 0.92)
Total anomalous pulmonary venous return	890	599	1.45 (1.30 to 1.60)
Patent ductus arteriosis	27392	25585	1.04 (1.02 to 1.06)
Other heart and circulatory sys anomalies	39287	34476	1.11 (1.09 to 1.13)
**Congenital anomalies of the digestive system**	34146	19545	1.70 (1.67 to 1.73)
Hirschsprung disease	1590	634	2.44 (2.23 to 2.67)
Biliary atresia	455	496	0.89 (0.79 to 1.01)
Esophageal atresia and tracheoesophageal fistula	1428	1194	1.16 (1.08 to 1.26)
Small intestinal atresia	1836	1884	0.95 (0.89 to 1.01)
pyloric stenosis	14781	3346	4.30 (4.14 to 4.46)
Rectal or large intestine atresia and stenosis	2704	2247	1.17 (1.11 to 1.24)
Other digestive anomalies	13504	11764	1.12 (1.09 to 1.14)
**Congenital anomalies of the genitourinary system**	62177	17662	3.42 (3.37 to 3.48)
Renal agenesis and hypoplasia	3491	2248	1.51 (1.43 to 1.59)
Obstructive genitourinary defects	24565	10912	2.19 (2.14 to 2.24)
Hypospadias	23834	80	289.8 (232.7 to 360.9)
Epispadias	960	12	77.7 (43.9 to 137.3)
Bladder exstrophy	145	114	1.24 (0.97 to 1.58)
**Congenital anomalies of the musculoskeletal system**	81783	69982	1.14 (1.13 to 1.15)
Limb reduction deformities			
Upper limb reduction deformities	1713	1396	1.19 (1.11 to 1.28)
Lower limb reduction deformities	858	760	1.10 (1.00 to 1.21)
Omphalocele	684	573	1.16 (1.04 to 1.30)
Clubfoot	6694	4252	1.53 (1.47 to 1.59)
Congenital hip dislocation	2302	7874	0.28 (0.27 to 0.30)
Diagphragmatic hernia	2026	1494	1.32 (1.23 to 1.41)
Gastroschisis	1942	1771	1.07 (1.00 to 1.14)
Craniosynostosis	18988	13941	1.33 (1.30 to 1.35)
Unspecified limb deformities	2542	2152	1.15 (1.09 to 1.22)
Other musculoskeletal anomalies	59745	49932	1.16 (1.15 to 1.18)
**Congenital anomalies of the integument**[b]	13168	11004	1.17 (1.14 to 1.19)
**Congenital anomalies of the ear, face, and neck**	22558	18539	1.18 (1.16 to 1.21)
Anotia_microtia	2043	1575	1.26 (1.18 to 1.35)
Other ear, face, and neck anomalies	21780	17931	1.18 (1.16 to 1.21)

**Oral clefts**	8939	7315	1.19 (1.15 to 1.23)
Cleft lip w/wo cleft palate	6195	3852	1.56 (1.50 to 1.63)
Cleft palate wo cleft lip	2764	3476	0.77 (0.74 to 0.81)
**Other unspecified congenital anomalies**	12131	10136	1.16 (1.13 to 1.20)

Supplementary Table 4. Hazard ratios from the mediation analysis for the association between sex and childhood cancer mediated by birth defect status; restricted to children age <5*

	Natural indirect effect HR (95% CI)†	Natural direct effect HR (95% CI)†	Total HR (95% CI)†	Proportion mediated, %‡	FDR-adjusted p-value for the natural indirect effect
**Any cancer**	1.04 (1.03 to 1.04)	1.06 (1.02 to 1.10)	1.10 (1.06 to 1.15)	42	<0.001
**Leukemia**	1.03 (1.03 to 1.04)	1.05 (0.98 to 1.13)	1.09 (1.01 to 1.16)	40	<0.001
Acute lymphoblastic leukemia	1.01 (1.01 to 1.02)	1.11 (1.02 to 1.20)	1.12 (1.04 to 1.22)	12	<0.001
Acute myeloid leukemia	1.10 (1.08 to 1.11)	0.89 (0.74 to 1.08)	0.98 (0.81 to 1.18)	--	<0.001
Other leukemia	1.09 (1.07 to 1.11)	0.90 (0.72 to 1.11)	0.98 (0.79 to 1.21)	--	<0.001
**Lymphoma**	1.03 (1.02 to 104)	1.50 (1.26 to 1.79)	1.54 (1.29 to 1.84)	8	<0.001
Hodgkin lymphoma	0.99 (0.98 to 1.01)	5.59 (2.16 to 14.4)	5.54 (2.14 to 14.3)	--	0.34
Non-Hodgkin lymphoma	1.03 (1.01 to 1.04)	1.26 (0.92 to 1.71)	1.29 (0.95 to 1.75)	11	0.005
Other lymphoma	1.03 (1.02 to 1.05)	1.48 (1.18 to 1.86)	1.53 (1.21 to 1.92)	9	<0.001
**Central Nervous System**	1.04 (1.04 to 1.05)	1.03 (0.94 to 1.13)	1.08 (0.98 to 1.18)	57	<0.001
Ependymoma	1.02 (1.00 to 1.03)	1.51 (1.12 to 2.04)	1.54 (1.14 to 2.08)	5	0.03
Medulloblastoma	1.05 (1.03 to 1.07)	1.52 (1.17 to 1.98)	1.60 (1.23 to 2.08)	13	<0.001
Astrocytoma	1.05 (1.04 to 1.06)	0.90 (0.78 to 1.04)	0.94 (0.81 to 1.08)	--	<0.001
Primitive neuroectodermal tumor	1.03 (1.01 to 1.06)	0.90 (0.58 to 1.39)	0.93 (0.60 to 1.43)	--	0.02
Other central nervous system	1.04 (1.03 to 1.05)	1.00 (0.86 to 1.17)	1.04 (0.89 to 1.21)	--	<0.001
**Peripheral Nervous System**	1.04 (1.04 to 1.05)	1.12 (1.00 to 1.26)	1.17 (1.04 to 1.31)	29	<0.001
Neuroblastoma	1.04 (1.04 to 1.05)	1.11 (0.99 to 1.24)	1.16 (1.03 to 1.30)	31	<0.001
Other peripheral nervous system	--	--	--	--	
**Retinoblastoma**	1.03 (1.02 to 1.04)	0.99 (0.83 to 1.18)	1.02 (0.85 to 1.21)	--	<0.001
**Renal**	1.03 (1.02 to 1.04)	0.82 (0.72 to 0.95)	0.85 (0.74 to 0.98)	--	<0.001
Nephroblastoma	1.03 (1.02 to 1.04)	0.80 (0.69 to 0.93)	0.82 (0.71 to 0.96)	--	<0.001
Other renal	1.03 (1.00 to 1.06)	1.13 (0.70 to 1.82)	1.17 (0.73 to 1.88)	21	0.04
**Hepatic**	1.12 (1.09 to 1.14)	1.34 (1.04 to 1.73)	1.50 (1.17 to 1.93)	32	<0.001
Hepatoblastoma	1.12 (1.09 to 1.14)	1.34 (1.03 to 1.73)	1.49 (1.15 to 1.93)	32	<0.001
Other hepatic	1.20 (1.09 to 1.32)	1.46 (0.49 to 4.37)	1.75 (0.59 to 5.21)	38	<0.001
**Bone**	1.01 (0.99 to 1.03)	0.98 (0.59 to 1.63)	0.99 (0.59 to 1.65)	--	0.42
Osteosarcoma	1.06 (0.96 to 1.18)	0.53 (0.13 to 2.24)	0.57 (0.14 to 2.38)	--	0.97
Ewing sarcoma	0.99 (0.97 to 1.02)	0.90 (0.43 to 1.86)	0.89 (0.43 to 1.85)	5	0.60
Other bone	1.01 (0.97 to 1.06)	1.37 (0.58 to 3.20)	1.38 (0.59 to 3.24)	5	0.54
**Soft tissue**	1.04 (1.03 to 1.05)	1.17 (1.00 to 1.37)	1.22 (1.04 to 1.43)	21	<0.001
Any rhabdomyosarcoma	1.02 (1.00 to 1.03)	1.28 (1.01 to 1.62)	1.30 (1.03 to 1.64)	7	0.01
Other rhabdomyosarcoma	1.02 (1.00 to 1.05)	1.43 (0.87 to 2.35)	1.46 (0.89 to 2.39)	6	0.11

Alveolar rhabdomyosarcoma	1.02 (0.99 to 1.05)	1.02 (0.57 to 1.82)	1.04 (0.58 to 1.86)	45	0.27
Embryonal rhabdomyosarcoma	1.01 (1.00 to 1.03)	1.30 (0.96 to 1.76)	1.32 (0.98 to 1.78)	5	0.08
Other soft tissue	1.06 (1.04 to 1.08)	1.10 (0.89 to 1.35)	1.16 (0.94 to 1.43)	40	<0.001
**Germ cell tumor**	1.10 (1.08 to 1.12)	1.03 (0.82 to 1.29)	1.13 (0.90 to 1.41)	81	<0.001
Extracranial germ cell tumor	1.16 (1.12 to 1.19)	0.41 (0.29 to 0.57)	0.47 (0.34 to 0.66)	--	<0.001
Gonadal germ cell tumor	1.04 (1.01 to 1.06)	6.80 (3.81 to 12.1)	7.04 (3.95 to 12.6)	4	0.004
Intracranial germ cell tumor	1.08 (1.03 to 1.13)	0.67 (0.38 to 1.21)	0.73 (0.40 to 1.30)	--	0.002
**Epithelial**	1.04 (1.01 to 1.08)	0.62 (0.39 to 0.99)	0.65 (0.41 to 1.03)	--	0.009
**Any other**	1.01 (0.99 to 1.03)	1.08 (0.68 to 1.70)	1.09 (0.69 to 1.72)	13	0.35

*Analyses are adjusted for birth year, state, maternal race/ethnicity, maternal education, maternal age, plurality.

† Empty cells are present when there were fewer of five cancer cases in the category.

‡ Empty cells are present when the proportion mediated is not estimable because the natural direct and indirect effects are in opposite directions.

Table 5. Hazard ratios from the mediation analysis for the association between sex and childhood cancer mediated by birth defect status; restricted to structural birth defects only*

	Natural indirect effect HR (95% CI)	Natural direct effect HR (95% CI)	Total HR (95% CI)	Proportion mediated, %†	FDR-adjusted p-value for the natural indirect effect
**Any cancer**	1.02 (1.02 to 1.03)	1.06 (1.03 to 1.10)	1.09 (1.05 to 1.12)	29	<0.001
**Leukemia**	1.01 (1.01 to 1.02)	1.12 (1.06 to 1.19)	1.13 (1.07 to 1.20)	9	<0.001
Acute lymphoblastic leukemia	1.01 (1.00 to 1.01)	1.16 (1.08 to 1.24)	1.16 (1.09 to 1.24)	4	<0.001
Acute myeloid leukemia	1.03 (1.02 to 1.04)	1.07 (0.90 to 1.27)	1.10 (0.93 to 1.31)	29	<0.001
Other leukemia	1.04 (1.03 to 1.06)	0.93 (0.76 to 1.12)	0.96 (0.80 to 1.17)	--	<0.001
**Lymphoma**	1.01 (1.01 to 1.02)	1.53 (1.37 to 1.71)	1.55 (1.39 to 1.73)	4	<0.001
Hodgkin lymphoma	1.00 (0.99 to 1.01)	1.49 (1.20 to 1.86)	1.49 (1.20 to 1.85)	--	0.64
Non-Hodgkin lymphoma	1.02 (1.01 to 1.03)	1.44 (1.20 to 1.74)	1.47 (1.22 to 1.77)	6	<0.001
Other lymphoma	1.02 (1.01 to 1.03)	1.63 (1.37 to 1.94)	1.67 (1.40 to 1.99)	6	<0.001
**Central Nervous System**	1.03 (1.02 to 1.03)	1.05 (0.98 to 1.13)	1.08 (1.01 to 1.16)	35	<0.001
Ependymoma	1.02 (1.00 to 1.03)	1.33 (1.04 to 1.69)	1.35 (1.06 to 1.71)	6	0.01
Medulloblastoma	1.04 (1.02 to 1.05)	1.58 (1.29 to 1.92)	1.64 (1.34 to 2.00)	9	<0.001
Astrocytoma	1.03 (1.02 to 1.04)	0.96 (0.86 to 1.07)	0.98 (0.88 to 1.10)	--	<0.001
Primitive neuroectodermal tumor	1.02 (1.00 to 1.04)	0.86 (0.59 to 1.26)	0.88 (0.60 to 1.29)	--	0.04
Other central nervous system	1.02 (1.02 to 1.03)	1.00 (0.90 to 1.12)	1.03 (0.92 to 1.14)	85	<0.001
**Peripheral Nervous System**	1.04 (1.03 to 1.05)	1.10 (0.98 to 1.23)	1.14 (1.02 to 1.27)	31	<0.001
Neuroblastoma	1.04 (1.03 to 1.05)	1.08 (0.97 to 1.21)	1.12 (1.01 to 1.26)	35	<0.001
Other peripheral nervous system	1.10 (1.00 to 1.21)	5.76 (1.30 to 25.6)	6.33 (1.43 to 28.0)	11	0.06
**Retinoblastoma**	1.02 (1.01 to 1.03)	1.00 (0.84 to 1.20)	1.02 (0.86 to 1.22)	82	<0.001
**Renal**	1.03 (1.02 to 1.04)	0.79 (0.69 to 0.90)	0.81 (0.71 to 0.92)	--	<0.001
Nephroblastoma	1.03 (1.02 to 1.04)	0.77 (0.67 to 0.88)	0.79 (0.69 to 0.91)	--	<0.001
Other renal	1.04 (1.01 to 1.07)	0.98 (0.64 to 1.51)	1.02 (0.67 to 1.56)	--	0.01
**Hepatic**	1.11 (1.09 to 1.13)	1.31 (1.03 to 1.66)	1.45 (1.14 to 1.84)	32	<0.001
Hepatoblastoma	1.11 (1.08 to 1.13)	1.33 (1.03 to 1.72)	1.48 (1.15 to 1.90)	30	<0.001
Other hepatic	1.14 (1.06 to 1.22)	1.10 (0.53 to 2.28)	1.26 (0.61 to 2.59)	60	<0.001
**Bone**	1.00 (1.00 to 1.01)	0.99 (0.81 to 1.22)	1.00 (0.81 to 1.23)	--	0.35
Osteosarcoma	1.00 (0.99 to 1.01)	0.98 (0.73 to 1.32)	0.99 (0.73 to 1.32)	--	0.99
Ewing sarcoma	1.00 (0.98 to 1.01)	1.02 (0.71 to 1.45)	1.01 (0.71 to 1.45)	--	0.65
Other bone	1.03 (1.00 to 1.07)	0.96 (0.56 to 1.63)	0.99 (0.58 to 1.68)	--	0.09
**Soft tissue**	1.03 (1.02 to 1.03)	1.10 (0.98 to 1.24)	1.13 (1.01 to 1.27)	22	<0.001
Any rhabdomyosarcoma	1.01 (1.00 to 1.02)	1.28 (1.06 to 1.56)	1.30 (1.07 to 1.57)	5	0.01
Other rhabdomyosarcoma	1.02 (1.00 to 1.04)	1.34 (0.89 to 2.02)	1.37 (0.91 to 2.05)	7	0.09

Alveolar rhabdomyosarcoma	1.01 (0.99 to 1.03)	1.01 (0.66 to 1.56)	1.02 (0.67 to 1.57)	45	0.35
Embryonal rhabdomyosarcoma	1.01 (1.00 to 1.02)	1.37 (1.06 to 1.77)	1.38 (1.07 to 1.79)	3	0.12
Other soft tissue	1.04 (1.03 to 1.05)	1.01 (0.87 to 1.17)	1.05 (0.90 to 1.21)	82	<0.001
**Germ cell tumor**	1.07 (1.05 to 1.08)	0.83 (0.70 to 1.00)	0.89 (0.75 to 1.06)	--	<0.001
Extracranial germ cell tumor	1.15 (1.11 to 1.18)	0.46 (0.33 to 0.63)	0.53 (0.38 to 0.73)	--	<0.001
Gonadal germ cell tumor	1.02 (1.01 to 1.04)	1.14 (0.88 to 1.49)	1.17 (0.90 to 1.52)	15	0.006
Intracranial germ cell tumor	1.05 (1.02 to 1.08)	1.02 (0.70 to 1.50)	1.07 (0.74 to 1.57)	69	<0.001
**Epithelial**	1.01 (1.00 to 1.02)	0.54 (0.45 to 0.64)	0.54 (0.46 to 0.65)	--	0.03
**Any other**	1.02 (1.00 to 1.04)	1.16 (0.79 to 1.71)	1.18 (0.80 to 1.75)	14	0.07

*Analyses are adjusted for birth year, state, maternal race/ethnicity, maternal education, maternal age, plurality.

†Empty cells are present when the proportion mediated is not estimable because the natural direct and indirect effects are in opposite directions.

Supplementary Figure 1. A simplified directed acyclic graph depicting the hypothesized causal association between males sex and risk of childhood cancer with birth defects as the mediator.

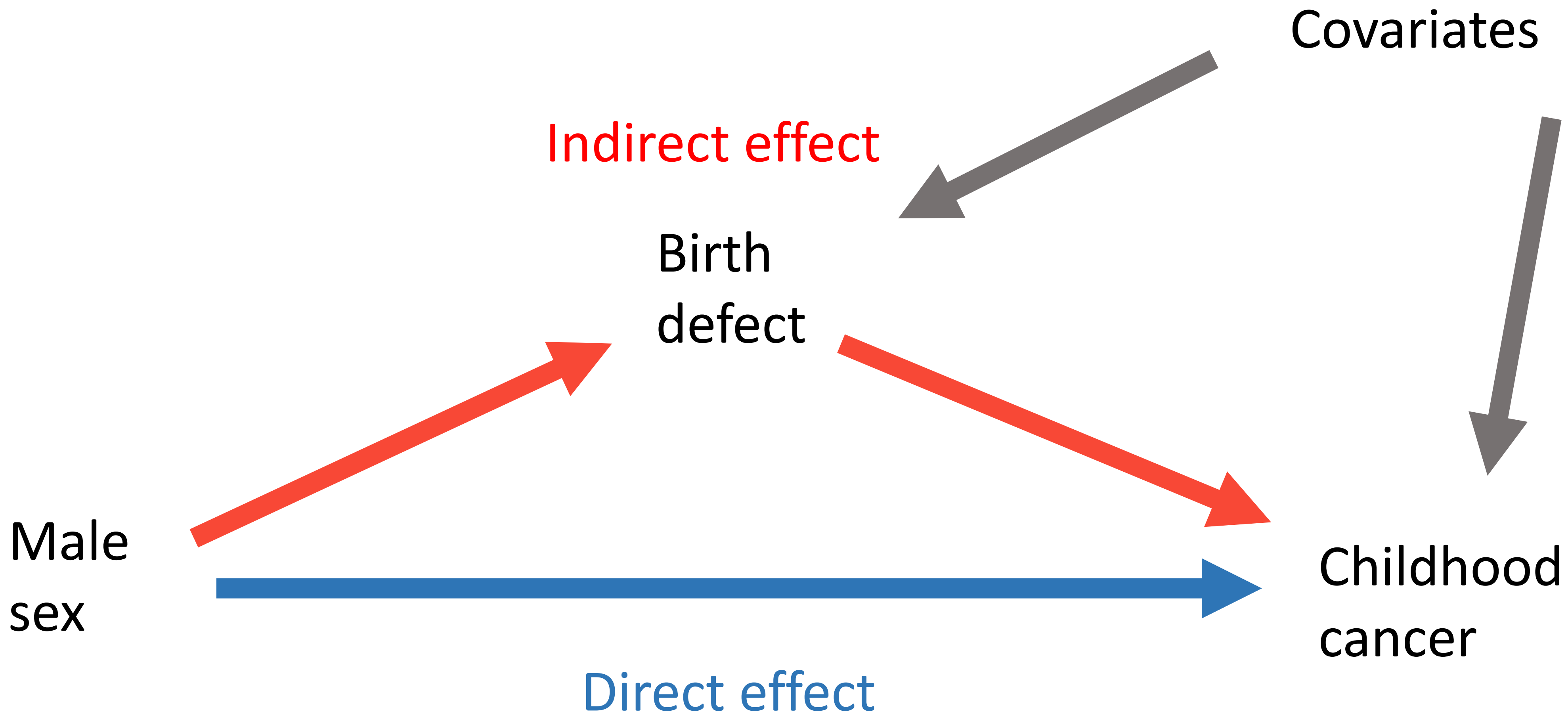

Supplement: pkaa052_Supplementary_Data [file pkaa052_supplementary_data.pdf]
